# 34 Using a National Burn Registry to Develop a Model for Risk-adjusted Length of Stay Benchmarking

**DOI:** 10.1093/jbcr/irac012.037

**Published:** 2022-03-23

**Authors:** Callie M Thompson, Palmer Q Bessey, Sara Higginson, Kimberly Hoarle, Naiwei Hsu, Darryl A Pardon, Bart D Phillips, Matthew H Phillips, Joan M Weber, Samuel P Mandell

**Affiliations:** University of Utah Health, Salt Lake City, Utah; New York-Presbyterian Hospital / Weill Cornell Medicine, New York, New York; UC San Diego Health Regional Burn Center, San Diego, California; American Burn Association, Chicago, Illinois; Torrance Memorial Medical Center, Torrance, California; BData, Inc, Louisville, Kentucky; BData, Inc, Edina, Minnesota; BData, Inc, Apex, North Carolina; Shriners Hospitals for Children Boston, Boston, Massachusetts; UT Southwestern, Parkland Regional Burn Center, Dallas, Texas

## Abstract

**Introduction:**

Length of stay (LOS) is a frequently reported outcome after a burn injury. Previous literature estimates LOS at 1 day per % total burn surface area (TBSA) but this varies considerably across patients & centers. LOS benchmarking will benefit individual burn centers as a way to measure their performance & set expectations for patients. We sought to create a nationwide, risk-adjusted model to allow for LOS benchmarking based on data from a national burn registry.

**Methods:**

Using data from a national burn registry, we queried admissions from 7/2015-6/2020 & identified 126,129 records with LOS data reported by 103 centers. We selected 23 predictor variables on the basis of completeness (min. 75% required) & clinical significance. Missing data were multiply imputed with a Bayesian Ridge Regression estimator. All statistics were calculated in Python using Numpy & Scikit-Learn libraries. Comparisons of unpenalized linear regression & Gradient boosted (CatBoost) regressor models were performed by measuring the R^2^ & concordance correlation coefficient (CCC) on the application of the model to the test dataset. The CatBoost model applied to bootstrapped versions of the entire dataset was then used to calculate O/E ratios for individual burn centers. Confidence intervals (CI) for O/E ratios were calculated using a normal distribution parametric model. Analyses were run on 3 cohorts: all patients, 10-20% TBSA, >20% TBSA.

**Results:**

The CatBoost model outperformed the linear regression model with a test R^2^ of 0.68 & CCC of 0.81 compared to the regression model with R^2^=0.52, CCC=0.70. The CatBoost was also less biased for higher & lower LOS durations. Due to the CatBoost model’s superiority in predicting the outcome, this model alone was used for O/E ratio calculations. The O/E ratio data from the model for all 3 cohorts are shown in Figure 1.

**Conclusions:**

Gradient boosted regression models provided greater model performance than traditional regression analysis. Using national burn data, we can predict LOS across contributing burn centers while accounting for patient & center characteristics, producing more meaningful O/E ratios. These models provide a risk-adjusted LOS benchmarking using a robust data source, the first of its kind, for burn centers.

